# KEADA: Identifying Key Classes in Software Systems Using Dynamic Analysis and Entropy-Based Metrics

**DOI:** 10.3390/e24050652

**Published:** 2022-05-06

**Authors:** Liuhai Wang, Xin Du, Bo Jiang, Weifeng Pan, Hua Ming, Dongsheng Liu

**Affiliations:** 1 School of Computer Science and Information Engineering, Zhejiang Gongshang University, Hangzhou 310018, China; 20020100080@pop.zjgsu.edu.cn (L.W.); 20020100014@pop.zjgsu.edu.cn (X.D.); lds1118@zjgsu.edu.cn (D.L.); 2 School of Engineering and Computer Science, Oakland University, Rochester, MI 48309, USA; ming@oakland.edu

**Keywords:** dynamic analysis, key classes, program comprehension, software network, one-order structural entropy

## Abstract

Software maintenance is indispensable in the software development process. Developers need to spend a lot of time and energy to understand the software when maintaining the software, which increases the difficulty of software maintenance. It is a feasible method to understand the software through the key classes of the software. Identifying the key classes of the software can help developers understand the software more quickly. Existing techniques on key class identification mainly use static analysis techniques to extract software structure information. Such structure information may contain redundant relationships that may not exist when the software runs and ignores the actual interaction times between classes. In this paper, we propose an approach based on dynamic analysis and entropy-based metrics to identify key classes in the Java GUI software system, called KEADA (identifying KEy clAsses based on Dynamic Analysis and entropy-based metrics). First, KEADA extracts software structure information by recording the calling relationship between classes during the software running process; such structure information takes into account the actual interaction of classes. Second, KEADA represents the structure information as a weighted directed network and further calculates the importance of each node using an entropy-based metric *OSE* (One-order Structural Entropy). Third, KEADA ranks classes in descending order according to their *OSE* values and selects a small number of classes as the key class candidates. In order to verify the effectiveness of our approach, we conducted experiments on three Java GUI software systems and compared them with seven state-of-the-art approaches. We used the Friedman test to evaluate all approaches, and the results demonstrate that our approach performs best in all software systems.

## 1. Introduction

With the continuous update of the software system, the functions of the software are increasing, and the structure is becoming more and more complicated. Generally, the high performance and reliability of a specific software is inseparable from the continuous maintenance of the software. However, due to the constant replacement of developers, new developers who want to maintain software must be familiar with the structure and function of software, which brings a lot of time overhead to software maintenance. Thus, how to maintain software efficiently has gradually received great attention from the filed of software engineering [1]. When maintaining unfamiliar software, developers should understand the structure and main functions of the software first, and this process accounts for 30% to 60% of the total time [2,3]. The traditional way for developers to understand software is through software development documents. However, the abilities of developers in a team are uneven, and the development documents may not be understood by all members easily. Thus, development documents alone are not enough for developers to achieve a quick understanding of the software.

Object-oriented (OO) software comprises many software entities, e.g., attributes, methods, classes, and packages. The running of the software is completed by the interaction among these entities. Classes are the basis of information encapsulation in object-oriented software and play a crucial role in the software system. Although there are many classes in a software system, only a few classes really play the important software functions and are treated as the key classes. In the literature, researchers have different views on the definition of key classes. Zaidman [4] believed that key classes have controlling, management functions in the system and manage other classes to realize the main functions of the software. Ding et al. [5] believed that some nodes frequently interact with other nodes in the software network, and the classes represented by these nodes were key classes that affected the structure and function of the whole software. If developers want to understand the software structure and main functions quickly [5,6,7], they must identify the key classes from the software. Thus, how to identify key classes from software systems has attracted extensive attention from developers.

In recent years, software is often mapped into a complex network [8,9,10,11,12,13,14,15], where the entities of the software (attributes, methods, classes, packages, etc.) are regarded as the nodes of the network, and the coupling relationships among the entities constitute the links of the network. Such a network model extracted from software is usually referred to as a *software network* [8,13,16,17]. Thus, the problem of identifying key classes in software can be transformed into the problem of extracting key nodes in software networks. Researchers have proposed some approaches to identify key classes, and most of them identified key classes by building static dependency networks [9,12,18,19,20,21,22]. Although these methods have made some progress in the identification of software key classes, there still exist some deficiencies: (i) *Lacking research work based on the dynamic analysis of the software:* The static analysis-based approaches built static dependency networks by analyzing the source code of the software without running the target software. The links in the network represent the coupling relationships that may exist among all entities in the software. These coupling relationships contain redundant relationships that may not actually exist when the software runs. (ii) *Ignoring the number of interactions between nodes:* In the static dependency network built by analyzing the source code of the software, the weights are calculated based on the complexity of the modules and the number of method calls; such a quantitative relationship cannot objectively reflect the real coupling strength between nodes when the software runs.

To fill this gap, we propose a key class identification approach in the Java GUI software system—KEADA (identifying KEy clAsses based on Dynamic Analysis and entropy-based metrics), which is based on dynamic analysis. First, our approach uses the automatic execution tool to run the function of Java GUI (Graphical User Interface) software, and it uses the bytecode rewriting tool to record the calling relationship between classes during the software running process. Second, we map the class call graph into a software network. The classes in the software are represented as the nodes in the network, the coupling relationships between classes are represented as the directed edges, and the coupling strengths between classes are represented as the weights on the edges. Finally, we use an entropy-based metric OSE (first-Order Structural Entropy) to quantify the importance of each class node in the network, and further rank all classes according to their importance. The top-ranked classes are regarded as the key classes identified by our approach. We evaluate our approach using three Java GUI software and compare the results with seven state-of-the-art approaches. The results demonstrate that our approach performs better than other approaches.

The main contributions of this work are summarized as follows:

- We propose a new approach to extract the software structure, which is implemented by automatic execution of the GUI software and dynamically tracing the execution of the software. The extracted information is further represented by a CCN (Class Call Network).
- We propose a new key class identification approach, which is based on the CCN and OSE metrics. Our approach is different from the existing approaches using structural information based on the static analysis of the target software.

The structure of the rest of this paper is organized as follows: Section 2 briefly summarizes the related work on key class identification. Section 3 introduces in detail the main steps of our approach to extract the software structure and identify key classes. Section 4 presents the experimental verification of our approach. Section 5 is the conclusion and future work.

## 2. Related Work

In the past few years, many approaches were proposed to identify key classes in software systems. These approaches can be roughly divided into two categories: approaches based on static analysis and approaches based on dynamic analysis.

The static analysis-based approaches analyze the source code of the software, extract the structure information of software and represent it as a software network, and finally use different metrics to measure the importance of the classes. Wang et al. [18] built a weighted software network by collecting three types of couplings in the system, i.e., the *inheritance* relationship, the coupling between classes and attributes, and the coupling between classes and methods. Based on the work of Wang et al., Steidl et al. [18,19] further considered two types of couplings, i.e., *interface implementation* and *return type*, to build directed, undirected software networks. The ICAN approach proposed by Pan et al. [9] considered the coupling of *parameter relations*, *member attributes*, and *local variables* when constructing a weighted software network. In a recent work, Du et al. [12] proposed a COSPA approach, which considered nine types of couplings when constructing weighted, directed software networks. In addition, researchers had also proposed many metrics to measure the importance of classes. *H*-index [23] was a metric for quantifying individual research results. Wang et al. [18] used the *h*-index and its variants to identify key classes. It was a lightweight and automated approach, and ensured the accuracy when identifying key classes. PageRank was an approach proposed by Brin and Page [24] to sort Web search results; it was also widely used to identify key classes by many researchers [18,19,20,21]. *K*-core is an approach used to find the closely related subgraph structure in a graph and can also be used to calculate the coreness of nodes in the network so as to measure the importance of the nodes. Recently, Pan et al. [22] considered the coupling direction and coupling strength in the network and proposed a generalized *k*-core decomposition approach to calculate the generalized coreness of nodes in the network. In addition, Li et al. [11] proposed the MinClass approach, which used the OSE metric to calculate the importance of classes. The COSPA approach was proposed by Du et al. [12], which used the *InDeg*, *OSE*, and *a*-index metrics to calculate the importance of each class. Then, they employed the Kemeny–Young approach to aggregate the three sequences returned by the three metrics to obtain a final ranking with the smallest total difference from the three sequences.

The dynamic analysis-based approaches constructed a software network by dynamically tracing the call information generated when the software is running, and then measured the importance of the nodes in the network so as to identify key classes. Zaidman and Demeyer [4] proposed a detection approach based on dynamic coupling and webmining. This approach collected the coupling information when the software is running, and used the HITS webmining algorithm to identify key classes in the software system. do Nascimento Vale and de Almeida Maia [25] used the Trace Extractor tool to collect the method-call relationships in the software, and built call trees of methods. Then, they compressed the call tree and deleted the same subtree, and finally classified the call tree to extract key subtrees and key classes.

In summary, the approaches based on static analysis may include the extra coupling relationships, which might not exist when the software runs. Worse still, they may ignore the number of object calls during the software’s real running when constructing the software network. The approaches based on dynamic analysis extracted the software structure information by obtaining the real interaction relationships when the software is running; however, the existing research work using dynamic analysis is still relatively weak [4,25]. The approaches based on the dynamic analysis and entropy-based metrics we proposed here can make up for this type of work to a certain extent. Specifically, our approach extracts method-call relationships when the software is running and further expresses it as class-level call information such that our approach does not produce redundant coupling relationships. Moreover, our approach considers the number of object calls during the software’s execution. It uses the number of calls between objects as the weight on the edges when constructing a software network.

## 3. The Proposed Approach—KEADA

**Figure 1:**
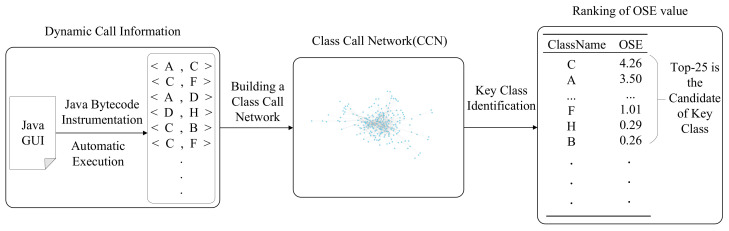
The framework of our KEADA approach.

The framework of our KEADA approach is shown in Figure 1. The KEADA approach mainly includes three parts: (i) Software structure information extraction: obtaining class-level call information according to method invocation when the software is running; (ii) software networks building: building weighted, directed software networks according to the method call information; and (iii) identifying key classes: employing the OSE metric to calculate the importance of each class and sorting them in descending order. We set a threshold, and the sorted classes within the threshold are regarded as the key classes identified by our approach.

### 3.1. Software Structural Information Extraction

The KEADA approach extracts class-level call information from the software system. It records the coupling relationships between classes in the Java GUI software through a bytecode rewriting tool, and this coupling relationship is generated by all method calls when the software runs. Java GUI is an event-driven program. GUI component objects generate various types of event messages according to user interaction. These event messages are captured by the event processing mechanism of the application, and then drive the message object to respond to it. Since the event-driven process of GUI is disordered and repeatable, disordered and repetitive driving a small number of events may produce a large number of paths, which increases the complexity of the event sequences. Manually clicking the button of the software to drive the program is no longer sufficient for large-scale software systems. We run all the functions in the target software by adapting the GUITAR tool [26]. Therefore, we divide the class-level call information extraction into two parts: (i) Java Bytecode Instrumentation; and (ii) Java GUI Automatic Execution.

#### 3.1.1. Java Bytecode Instrumentation

In order to obtain the coupling relationship of the classes generated by method calls during the running of the software, we need to insert a marker in each method of each class to record the call path. Javassist, a bytecode rewriting tool, can help us fulfill this purpose. Before describing how we get class-level call information, we need to explain some terms, as follows.

Java Instrumentation: Instrumentation [27] refers to an agent program which is independent of the application program, and it can be used to monitor and assist the application program that is running on the JVM. Instrumentation provides a method called “premain”, which specifies the agent program through the “-javaagent” parameter. The agent program started before the main method, and it can load all the classes required by the software to run.

First of all, we filter all classes before the main program is started through the Java Instrumentation mechanism, because these classes may contain classes in the library required for the software to run. We only need to reserve the classes of Java GUI software, which can be completed by identifying the package name. Then, we use the bytecode rewriting tool Javassist to insert a marker into all method bodies of the reserved class. This marker helps us record two pieces of information: the class of this method and the class which calls this method, and they can be obtained in the information of thread stack. In this way, we can obtain the class-level call information generated by calling all methods during the software’s running. This call information is saved as <Caller,Callee>. Note that our approach extracts the structure information following two steps, i.e., (i) it captures the method call when the system is running, and (ii) it maps the couplings between methods to the couplings between classes. Thus, our approach cannot capture the couplings with interfaces and enums, as they do not contain a method body or even do not have any method. From here on, if not mentioned, our classes do not contain interfaces and enums.

#### 3.1.2. Java GUI Automatic Execution

**Figure 2:**
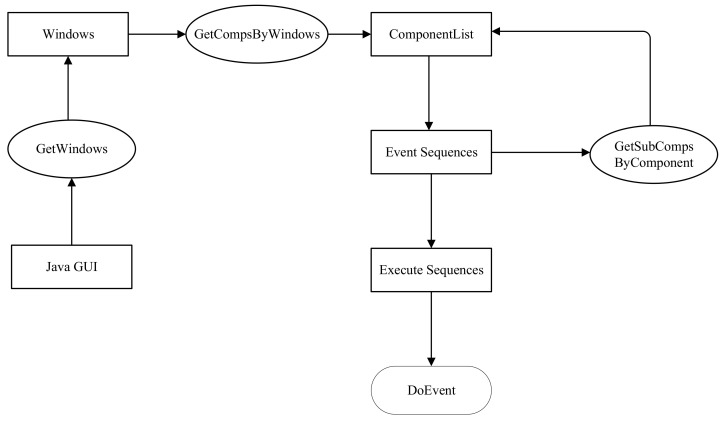
The framework of automatic execution.

**Figure 3:**
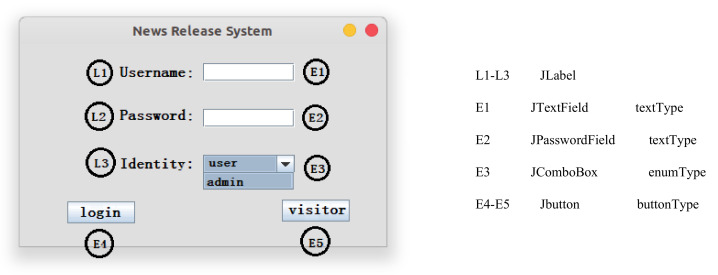
A Java GUI example.

Through the above operations, we can obtain the class-level call information generated by calling all methods during the software’s running. We use automatic execution instead of manually clicking the button to execute all the functions of the program. The main steps are shown in Figure 2. First, it needs to start the program to get the window of the Java GUI software. Then, we obtain the component list through the windows, and put the event components in the component list into the event sequences. There may be some components that contain sub-components; thus, we need to use recursive methods to obtain all components. Finally, we execute the event sequences according to the event type, and obtain the dynamic call graph of the class. Figure 3 is a simple example of a Java GUI. We will explain each step in more detail in conjunction with Algorithm 1.

Step 1: Obtain event sequence. We use the reflection mechanism to start the News Release System on the left in Figure 3. Then, we obtain all the components (i.e., E1–E5 and L1–L3 in Figure 3) by the login windows of the News Release System. As there are some components (JMenu, etc.) that contain subcomponents, we use recursive methods (function GetAllComponents in Algorithm 1) to obtain all components. These components include interactive event components (i.e., E1–E5) and non-interactive label components (i.e., L1–L3). We distinguish these components and store the interactive event components in the event sequence.

Step 2: Generate execution sequences. As there are many types of components in the event sequence, we divide them into three cases. Components of text type (JTextfield, JPasswordField, JTextArea, etc.) are directly added to the execution sequences. Since the input data of text type cannot be generated automatically, we need to create a configuration file that stores the input data of all components of the text type. For components of the enum type (JComboBox, ButtonGroup, etc.), we need to get the number of event values of the component. Then, it combines other components according to different numbers and adds them to the execution sequence. As shown in Figure 3, E3 has two event values. We add it with the combination of E1, E2, and E4 to the execution sequence to obtain the following sequence: E1, E2, E3 (value1), E4, E1, E2, E3 (value2), E4, etc. For components of the button type, we add them directly to the execution sequences. Please note that the execution path may be missed by this operation, and these paths need to be added to the execution sequences manually.

Step 3: Run the execution sequence. The components in the execution sequences are stored as component objects, which can be downcast into specific component types. Components of the text type have a method named “setText”. We obtain the input data information corresponding to the component in the configuration file, and then add the input data to the text box through the “setText” method. Since there are many components of the enum type, we use the enum type in Figure 3 as a case. In Figure 3, E3 uses the “setSelectedIndex” method to set the selected index to achieve automatic execution. Components of the button type have a “doClick” method through which we can complete the automatic execution of the button type.

The Java GUI program can perform most of the functions automatically through such operations. If there are multiple windows in the program or other windows started after some components are executed (the visitor program will be opened after E5 is driven in Figure 3), the components of these windows can be executed automatically using the same steps. Although our automatic execution program is not complete (cannot contain all types of component in Swing/AWT), we can automatically execute the components commonly used in the GUI software. For components that are not contained, you can manually click to drive events to achieve their functions.
**Algorithm 1** Java GUI automatic execution**Require:** Login.class1: /*Use reflection mechanism to start the program*/2: clazz←Login.class3: main←clazz.getMethod(“main”, args)4: main.invoke(args)5: /*Step1:Get event sequence*/6: MainWindows← JFrame.getWindows[0] //Get the login window7: windows← (Container)MainWindows //Upcast to Container objecet8:  9: **function**GetAllComponent(windows) //Get all components10:     allComponents←windows.getComponents()11:     **for** com in allComponents **do**12:         container← (Container)com13:         allComponents.add(GetAllComponent(container))14:     **end for**15:     **return** allComponents16: **end function**17:  18: **for**com in allComponents **do**19:     **if** com.getAccessibleContext().getAccessibleAction()!=null **then**20:         eventSequence.add(com) //Store interactive events in the event sequence21:     **end if**22: **end for**23:  24: /*Setp2:Generate execution sequences*/25: **function**GeneExecSequence(eventSequence)26:     **for** com in eventSequence **do**27:         **if** com is textType **then**28:            //textType refers to JTextField, JPasswordField, JTextArea, etc.29:            Join the execution sequence and store input data to configuration file30:         **end if**31:         **if** com is enumType **then** //enumType refers to JComboBox, JCheckBox, etc.32:            Get the number of event values, and combine other components to33:            join the execution sequence34:         **end if**35:         **if** com is buttonType **then** //buttonType refers to JMenu, JButton, etc.36:            Join the execution sequence37:         **end if**38:     **end forreturn** executeSequence39: **end function**40:  41: /*Setp3:Run the execution sequence*/42: **function**ExecSequence(executeSequence)43:     **for** com in executeSequence **do**44:         **if** com is textType **then**45:            Get text from the configuration file46:            com.setText(text)47:         **end if**48:         **if** com is enumType **then**49:            com.setSelectedIndex(index)50:         **end if**51:         **if** com is buttonType **then**52:            com.doClick()53:         **end if**54:     **end for**55: **end function**

### 3.2. Software Network Definition

In this work, the obtained class-level call information is represented by a *class call network*, which is formally defined as follows.

**Definition** **1.** *Let CCN=(N,E) be a class call network, where N is a set of nodes, representing the classes in a piece of Java GUI software; $E=\{<N_{i},N_{j}>|N_{i},N_{j}\in N\}$ is a set of directed edges, where $<N_{i},N_{j}>$ denote calls from node $N_{i}$ to node $N_{j}$, and each directed edge is assigned a weight $W<N_{i},N_{j}>$ to represent the number of calls between a pair of classes.*

**Figure 4:**
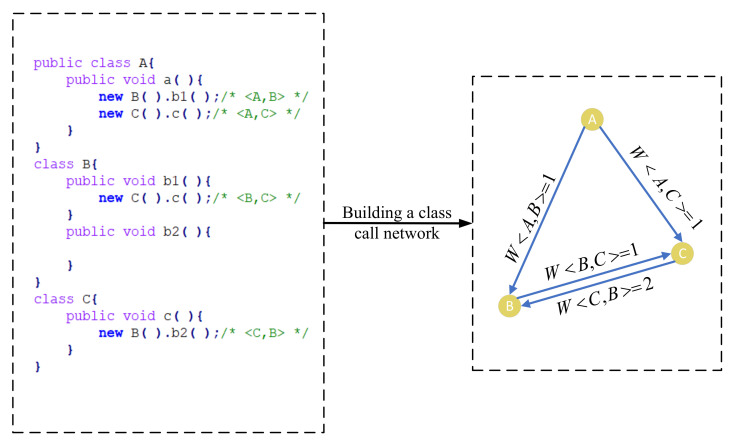
A simple code snippet (the left part) and its corresponding CCN (the right part).

We use a simple example in Figure 4 to illustrate our network. When the method “*a*” of Class *A* is called, the interactive relationship between Class *A* and Class *B* is W<A,B>=1, the interactive relationship between Class *B* and Class *C* is W<B,C>=1 and W<C,B>=2, and the interactive relationship between Class *A* and Class *C* is W<A,C>=1. Such interactive relationships are further mapped to a network, which is shown in the right part of Figure 4.

### 3.3. OSE (First-Order Structural Entropy) Metric

In the CCN of a specific software system, the weight on the edge represents the call intensity between the two classes, and the calls between two classes are directional. Therefore, we need to consider the calling direction and strength between classes when choosing the metric to measure the importance of classes. Li et al. [11] proposed an entropy-based metric OSE to measure class importance. It is reported that when using *OSE* to identify key classes, they only need to check a very small number of top-ranked classes. *OSE* considered four attributes of classes that may affect the importance measurement of classes, that is, the number of neighbour nodes, the weight on edge, the edge weight distribution connected to neighbours, and the importance of neighbour nodes. In the CCN, a node represents a class, the number of neighbour nodes represents the number of classes interacting with this class, and the weight on the link with neighbour nodes represents the number of interactions with other classes. Different classes have different weight distributions on the link with their neighbour nodes, and their neighbour nodes have different importance. *OSE* considered the influence of these factors when measuring the importance of class; thus, *OSE* was suitable for identifying key classes. OSE is defined as

(1) $$\begin{array}{c} P_{xw}=\frac{w_{xw}/w_{max}}{\sum_{y\in in(w)}w_{yw}/w_{max}},x,w\in N, \end{array}$$

(2) $$\begin{array}{c} h_{w}=(1-\sum_{x\in in(w)}\left(P_{xw}InP_{xw}\right))\sum_{x\in in(w)}w_{xw}/w_{max},w\in N, \end{array}$$

(3) $$\begin{array}{c} OSE_{w}=h_{w}+\sum_{v\in in(w)}(\frac{w_{vw}/w_{max}}{\sum_{u\in on(v)}w_{vu/w_{max}}}h_{v}),w\in N, \end{array}$$

where *N* denotes the set of class nodes in the CCN; *x* and *w* denote two class nodes in *N*; $w_{xw}$ denotes the weight on the edge *x* to *w*; $w_{max}$ denotes the maximum weight on all edges of CCN; in(w) denotes the in-neighbours of class *w*, on(w) denotes the out-neighbours of class *w*; and $OSE_{w}$ denotes the OSE value of class *w*.

Formula (1) is used to calculate $P_{xw}\left(x,w\in N\right)$, which considers the weight on edge (i.e., $w_{xw}$) and the weight linked with neighbour nodes (i.e., $\sum_{y\in in(w)}w_{yw}/w_{max}$). $w_{xw}/w_{max}$ normalizes the weight on the edge, and in(w) considers the influence of the number of neighbour nodes in measuring the importance. Formula (2) is used to calculate $h_{w}\left(w\in N\right)$, which takes into account the weight edge distribution on the neighbour node (i.e., $\sum_{x\in in(w)}(P_{xw}InP_{xw}$) when measuring the importance of classes. $\sum_{x\in in(w)}w_{xw}/w_{max}$ denotes the sum of the weight linked with neighbour nodes. Formula (3) is used to calculate the OSE value of the node by combining the importance of this node (i.e., $h_{w}$) and the importance of neighbour nodes (i.e., $\sum_{v\in in(w)}\left(\frac{w_{vw}/w_{max}}{\sum_{u\in on(v)}w_{vu/w_{max}}}h_{v}\right)$).

**Figure 5:**
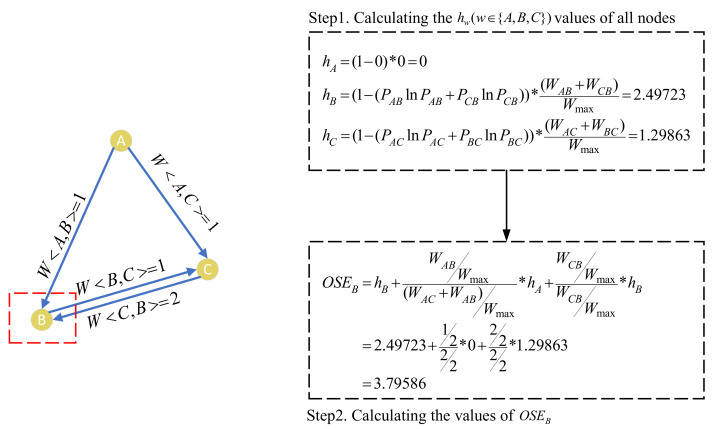
Illustration of the process to compute OSE.

Figure 5 shows a simple example to exhibit how to calculate the *OSE* of nodes in a network. The left part of Figure 5 shows a CCN, and the right part shows the process to calculate the OSE value of node *B* as an example. First, we calculate the $P_{xw}\left(x,w\in \left\{A,B,C\right\}\right)$ value of each edge in the CCN. We get $w_{AC}=1,w_{AB}=1,w_{BC}=1,$ and $w_{max}=w_{CB}=2$ from the left part of Figure 5, and then take it into formula (1) to calculate $P_{AB}=\frac{w_{AB}/w_{max}}{w_{CB}/w_{max}+w_{AB}/w_{max}}=\frac{1/2}{2/2+1/2}=0.33333.$ In the same way, we get $P_{AC}=0.5$, $P_{BC}=0.5$ and $P_{CB}=1.5$. Second, we calculate $h_{w}\left(w\in N\right)$ in the CCN by combining the weight edge distribution on the neighbour node. We take $P_{xw}$ (x,w∈ {A, B, C}) value into formula (2) to calculate the $h_{w}$ (*w*∈ {A, B, C}) value of each node in the CCN, and we obtain $h_{A}=0,h_{B}=2.49723$, and $h_{C}=1.29863$ (the detailed calculation process is demonstrated in Setp 1 of Figure 5). Finally, we normalize the *h* value of the neighbour node and combine it with the *h* value of this node to calculate the *OSE* value of the node. $h_{w}$ and $P_{xw}$ are brought into formula (3) to calculate the value of $OSE_{B}$, which is equal to 3.79586 (the detailed calculation process is demonstrated in Setp 2 of Figure 5).

### 3.4. Key Class Identification

We calculate the *OSE* value of each class node in the CCN and sort all class nodes in descending order according to the *OSE* value. After sorting, we will select some classes within the threshold as our candidate key classes; however, how many classes should be considered as the key class candidates? It is important to know that different software has unequal scales. In the literature, researchers usually used 15% as a threshold to find the candidate set of the key classes, i.e., the 15% top-ranked classes in the list are the potential key classes. However, even when such a threshold is applied, the obtained candidate set of the key classes might be still very large. Thus, in this work, we take the top-25 classes as candidate key classes. The rational for choosing the top-25 classes is twofold: (i) Brain et al. [24] reported that the number of key classes in software systems are not proportional to the size of the software, and the average number of key classes is between 20 to 30; and (ii) using top-25 as the threshold has also been applied in some recently published work [11,12].

## 4. Empirical Study

In this section, in order to verify the effectiveness of our KEADA approach on key class identification, we used three Java GUI software to evaluate it.

### 4.1. Research Questions

In this work, we focus on the following research question:

RQ: *Is our KEADA approach, based on dynamic analysis and entropy-based metrics, better than the existing approaches based on static analysis?* In this work, we apply our approach to three Java GUI software and compare it with other seven baseline approaches in the literature to verify the effectiveness of our approach.

### 4.2. Subject Systems

There are sixteen open-source software as subject systems in the existing work of identifying key classes. Since our approach can only identify key classes of GUI software, there are six software left for selection. Our approach is not suitable for GUI software with too many text-type components in the window, so we use three open-source Java GUI software as our subject systems. The main reason why we use these systems is that they contain the true key classes in each system, and thus we can use them to compute the metric values, such as *Recall* and *Ranking Score*. If our approach is applied to other systems, then one should build the *gold set* (i.e., true key classes); otherwise, she cannot compute the values of evaluation metrics. Table 1 gives the details of the three software, including the system name, version number, number of classes (#classes), number of methods (#methods), and URLs to download the corresponding software system. Note that #classes does not contain the number of interfaces and enums.

### 4.3. Evaluation Metrics

We use *Recall* [28,29,30] and *Ranking Score* as metrics to evaluate the effectiveness of different approaches on key class identification. *Recall* is the ratio of the number of key classes identified by a specific approach to the total number of true key classes. Ranking Score is the average position of key classes in the sorting list of key classes identified by a specific approach.

*Recall* is formally defined as

(4) $$\begin{array}{c} Recall=\frac{TP}{TP+FN} \end{array}$$

where TP represents the number of classes in the reference set that are identified by a specific approach as key classes, and FN indicates the number of key classes not successfully identified by a specific method.

(5) $$\begin{array}{c} Ranking\;Score=\frac{\sum_{x\in (\mathrm{TPSet}+\mathrm{FNSet})}\mathrm{Pos}\left(x\right)}{\mathrm{TP}+\mathrm{FN}} \end{array}$$

where TP+FN indicates the total number of key classes in a piece of software, TPSet+FNSet indicates the set of key classes in a piece of software, and Pos(x) represents the position of the key class *x* in the sorted list of classes returned by a specific approach.

### 4.4. Baseline Approaches

As we have reviewed in the section of Related Work, there are many approaches to measure the importance of class nodes in software networks. In order to compare our KEADA approach with other approaches, we select seven static analysis-based approaches as our baseline approaches, i.e., CONN-TOTAL [31], PageRank [20,21,24], ICOOK [22], a-index [18], h-index [18], Coreness [32], and MinClass [11]. We did not select other approaches as the baseline approaches because we lacked enough information to replicate their work. These approaches are briefly described as follows.

- CONN-TOTAL: This calculates the degree of a specific class as its importance. Numerically, it equals to the sum of the in-degree and out-degree of the class.
- PageRank: This calculates the PageRank value of a specific class as its importance.
- ICOOK: This employs a generalized k-core decomposition to calculate the generalized coreness of each class as its importance.
- Coreness: This uses k-core decomposition to calculate the coreness of each class as its importance.
- MinClass: This uses OSE of each class node in a CCN as its importance.

### 4.5. Results and Analysis

We performed a series of experiments to validate the effectiveness of our approach when compared with the other seven approaches.

***RQ*** *: Is our KEADA approach, based on dynamic analysis and entropy-based metrics, better than the existing approaches based on static analysis?*

After performing a series of steps, such as extracting the class call information of software, defining the class call network, and computing the importance of the class nodes, we have obtained a sorted list of classes. Then, we choose the top-25 highly ranked classes as the candidate set of the key classes identified by our KEADA approach.

Table 2 shows the results of comparison between our approach and the other seven baseline approaches, and the details are shown in Table 3, Table 4 and Table 5. The “Key classes” column lists all the key classes in the corresponding software, and the “Identified” column indicates whether the key classes can be successfully identified by our approach. In this column, “✓” means that our approach can effectively identify the key class, while “×” means that it has not been successfully identified. It is worth noting that the “N/A” appears in this column, which means that our approach does not extract this class from the software structure. By checking the running of the software, we found that the cause of this situation was that these key classes are interfaces or enums. Our method of extracting structural information is to obtain the calling information in the method body of the class. Still, there is no method or method body in interfaces and enums. The “Position” column shows the position information of the key class in the ordered class list. Since some of the classes are missing from the class list, we set the “Position” of these classes to “-” in this column. *Recall* is an important indicator for evaluating our approach. Note that there are some class nodes with a same “Position” value, and thus cannot be sorted. If this situation occurs, we adopt the average position as their final “Position” value. For example, when using the *Coreness* to calculate the importance of the class node, the positions of classes “ToDoList” and “Designer” are 4 and 5, respectively, in the class list. However, in fact, they have the same *coreness* value and actually have the same probability (i.e., 50%) to be ranked at positions 4 and 5. Thus, in this work, their final “Position” values are both (4 + 5)/2 = 4.5.

From Table 3, we observe that, when we apply the KEADA approach to software “Argo UML”, the *Recall* of our approach reaches 75.00%. It can be found that the *Recall* is higher than other approaches. It means that our approach has better performance on identifying key classes in this software.

As shown in Table 4, in JHotDraw, our KEADA approach has more superior performance. It can be observed that our approach can achieve the best performance (the *Recall* reaches 100%). In other words, our approach can retrieve all the key classes in the top-25 ranked classes. Since we are applying an entropy-based metric *OSE* to the software network built by dynamically tracing the software execution, we will focus on the comparison with the MinClass approach, which also uses the *OSE* metric to calculate the importance of classes in the software network built by static analysis. The result demonstrates that our approach is significantly better than MinClass; the *Recall* is increased by 33.33%. Moreover, comparing our approach with other six approaches, our approach still performs better according to *Recall*.

We apply our approach to Maze, and the results are shown in Table 5. We can observe that our approach can retrieve 11 key classes among the top-25 ranked classes. Although our approach has a slightly lower *Recall* value than that of CONN-TOTAL, ICOOK, Coreness, and MinClass, it is indeed superior to a-index, PageRank, and h-index. Comparing the value of *Ranking Score*, we can find that the average position of our key class is lower than that of other six approaches, only higher than that of ICOOK. It shows that, in addition to the ICOOK approach, compared with the other six approaches, the key classes in the class list obtained by our approach are ranked higher.

**Figure 6:**
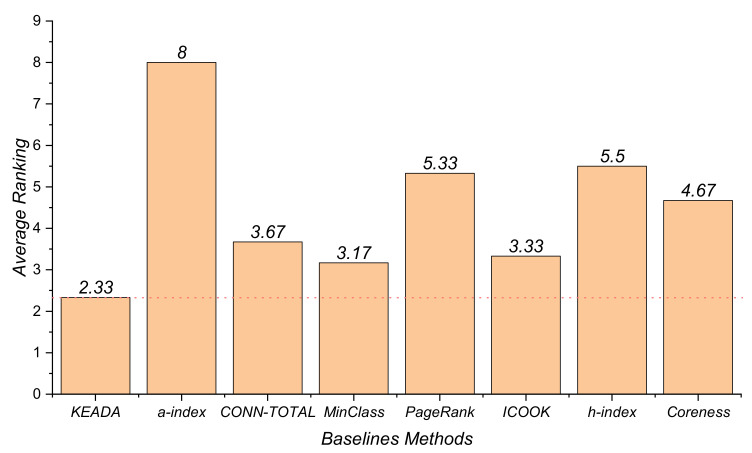
Results of the average ranking of the Friedman test.

From the above experimental results, it can be found that different approaches perform differently on the three software systems. Thus, we were unable to find one that has the best performance across all the software systems. In this work, in order to compare the performance of the different approaches in all the software systems, we use the Friedman test. Figure 6 shows the results of the average ranking of the baseline approaches in terms of the *Recall* metric. The smaller the value is, the better the performance of the approach. The results in Figure 6 demonstrate that the average ranking of KEADA is smaller than that of the remaining seven approaches. Even though the MinClass also performs well, there is still a gap to our KEADA, as evidenced by the fact that the average ranking of the KEADA is lower than that of the MinClass. Therefore, our answer to the RQ is that our approach does perform better than other baseline approaches on the key class identification according to the results of the Friedman test.

### 4.6. Threats to Validity

Although our approach does perform better on the key class identification of Java GUI software, it also faces many threats.

The internal threat that affects the effectiveness of our work is that our approach cannot deal with interface and enum when extracting the class-level call information. This is because we obtain the call information by inserting the marker in the method body, and there is no method body in the methods of interfaces and no method in the enums. This threat is increased from the interface that has default and static methods in Java 8. However, we found by calculating the *OSE* value of all classes that if the interface or enum is a key class, then the OSE value of the implementation class of the interface is ranked high. It also indirectly shows that our approach can identify the key classes of the interface. In future work, we will consider an objective method to deal with the interface and enum.

The external threat is that our approach can only identify key classes in Java GUI software. It cannot be extended to GUI software in other programming languages, or multilingual GUI software that uses Java as the main language. We will use other programming languages to replicate our work in the future.

The reliability threat is that our approach is not suitable for Java GUI software with a large number of text-type components, which requires enough and correct text information to make most functions of the program run. In future work, we will collect the textual information that makes the GUI software of text-type components run. In this way, this threat can be mitigated.

## 5. Conclusions and Future Work

This approach is based on dynamic analysis, which extracted software structure information by obtaining the interaction relationships when the software is running. Therefore, if the structure information extracted in this way is accurate, it is necessary to run all the software functions as much as possible. The running of Java GUI software is to drive different types of components, and KEADA can obtain these components and make them drive to run all functions of the software. However, for other Java software, KEADA does not make the program work. In addition, Java GUI software with a large number of text-type components needs enough correct text information to make the most of the program’s functions run. Therefore, for Java software without a graphical interface and Java GUI software with a large number of text-type components, it is not suitable to use KEADA to identify key classes. For other types of Java GUI software, the key classes identified by KEADA perform better than the static approaches.

KEADA provides an idea for identifying key classes based on dynamic analysis, but it still has some shortcomings. In view of the shortcomings of our method, we establish a set of future work routes: (1) Since our method cannot identify interfaces and enums, we will find an objective method for identifying these types of key classes; (2) our approach can currently only be used to identify key classes for Java GUI software, thus, we will replicate this work on other software in the Java programming language; and (3) improving our approach of identifying key classes to increase its performance.

## Figures and Tables

**Table 1:** Statistics of the subject systems.

System	Version Number	#Classes	#Methods	URLs (accessed on 27 March 2020)
ArgoUML	0.9.5	1038	6178	https://argouml-tigris-org.github.io/
JHotDraw	6.0b.1	217	6737	https://sourceforge.net/projects/jhotdraw/
Maze	1	71	563	https://code.google.com/p/maze-solver/

**Table 2:** Comparison of the results obtained by different approaches.

Evaluation Metrics	ArgoUML		JHotDraw		Maze	
	Recall	Ranking Score	Recall	Ranking Score	Recall	Ranking Score
KEADA	**75.00%**	31.25	**100%**	**4**	45.83%	27.19
a-index	0%	264.00	0%	180.22	29.63%	35.63
CONN-TOTAL	66.67%	**26.08**	66.67%	16.11	48.15%	27.41
MinClass	66.67%	27.33	66.67%	26.67	51.85%	27.39
PageRank	58.33%	29.08	66.67%	23	40.74%	34.39
ICOOK	33.33%	85.83	88.89%	12.67	**55.56%**	**26.59**
h-index	41.67%	88.17	66.67%	20.33	44.44%	28.24
Coreness	41.67%	67.83	66.67%	29.44	48.15%	38.57

The bold denotes the best performance value for the Recall and Ranking Score of each software.

**Table 3:** Comparison of the results obtained by different approaches when applied to Argo UML.

Key Classes	KEADA		a-Index		CONN-TOTAL	
	Identified	Position	Identified	Position	Identified	Position
CrUML	✓	2	×	166.5	✓	5
ToDoList	✓	15	×	255	×	28
Designer	✓	8	×	162	✓	1
ToDoItem	✓	4	×	299	✓	7.5
ProjectBrowser	×	41	×	284.5	✓	2
Argo	×	162	×	321	✓	18
History	N/A	-	×	282	×	83
Project	N/A	-	×	264	✓	6
ControlMech	N/A	-	×	147	×	96
Critic	✓	1	×	197	✓	3
Configuration	✓	17	×	268	×	39
Wizard	N/A	-	×	522	✓	24.5
Recall (%)	**75.00%**		0%		66.67%	
Ranking score	31.25		264.00		**26.08**	
**Key Classes**	**MinClass**		**PageRank**		**ICOOK**	
	**Identified**	**Position**	**Identified**	**Position**	**Identified**	**Position**
CrUML	✓	3	✓	9	×	49
ToDoList	✓	13	×	79	✓	25
Designer	✓	1	✓	6	✓	22
ToDoItem	✓	6	✓	13	×	32
ProjectBrowser	✓	7	✓	2	✓	23
Argo	✓	12	✓	21	×	33
History	×	55	×	49	×	234
Project	✓	21	✓	5	✓	24
ControlMech	×	142	×	52	×	393
Critic	✓	5	✓	8	×	37
Configuration	×	32	×	68	×	58
Wizard	×	31	×	37	×	100
Recall (%)	66.67%		58.33%		33.33%	
Ranking score	27.33		29.08		85.83	
**Key Classes**	**h-Index**		**Coreness**			
	**Identified**	**Position**	**Identified**	**Position**		
CrUML	×	49.5	×	43.5		
ToDoList	×	30	✓	14		
Designer	✓	16.5	✓	14		
ToDoItem	✓	16.5	✓	14		
ProjectBrowser	✓	1	✓	14		
Argo	✓	16.5	×	43.5		
History	×	357.5	×	239.5		
Project	✓	2	✓	14		
ControlMech	×	357.5	×	239.5		
Critic	✓	30	×	43.5		
Configuration	×	90.5	×	91		
Wizard	×	90.5	×	43.5		
Recall (%)	41.67%		41.67%			
Ranking score	88.17		67.83			

The bold denotes the best performance value for the Recall and Ranking Score of Argo UML.

**Table 4:** Comparison of the results obtained by different approaches when applied to JHotDraw.

Key Classes	KEADA		a-Index		CONN-TOTAL	
	Identified	Position	Identified	Position	Identified	Position
CompositeFigure	✓	1	×	179	×	42.5
StandardDrawingView	✓	3	×	110	✓	16.5
DrawApplication	✓	8	×	157	✓	5
Figure	N/A	-	×	256	✓	2
DrawingEditor	N/A	-	×	279.5	✓	6
Drawing	N/A	-	×	159	✓	7
Tool	N/A	-	×	174	×	27.5
DrawingView	N/A	-	×	166.5	✓	1
Handle	N/A	-	×	141	×	37.5
Recall (%)	**100%**		0%		66.67%	
Ranking score	**4**		180.22		16.11	
**Key Classes**	**MinClass**		**PageRank**		**ICOOK**	
	**Identified**	**Position**	**Identified**	**Position**	**Identified**	**Position**
CompositeFigure	×	71	×	43	✓	16
StandardDrawingView	×	84	✓	5	✓	5
DrawApplication	×	29	✓	6	✓	9
Figure	✓	1	✓	3	✓	1
DrawingEditor	✓	5	✓	9	✓	10
Drawing	✓	6	✓	12	✓	4
Tool	✓	20	×	65	✓	22
DrawingView	✓	2	✓	2	✓	2
Handle	✓	22	×	62	×	45
Recall (%)	66.67%		66.67%		88.89%	
Ranking score	26.67		23		12.67	
**Key Classes**	**h-Index**		**Coreness**			
	**Identified**	**Position**	**Identified**	**Position**		
CompositeFigure	×	47	✓	22.5		
StandardDrawingView	✓	11	✓	22.5		
DrawApplication	✓	24.5	×	44		
Figure	✓	1	✓	5		
DrawingEditor	✓	15	✓	22.5		
Drawing	✓	6.5	✓	22.5		
Tool	×	37.5	×	44		
DrawingView	✓	3	✓	22.5		
Handle	×	37.5	×	59.5		
Recall (%)	66.67%		66.67%			
Ranking score	20.33		29.44			

The bold denotes the best performance value for the Recall and Ranking Score of JHotDraw.

**Table 5:** Comparison of the results obtained by different approaches when applied to Maze.

Key Classes	KEADA		a-Index		CONN-TOTAL	
	Identified	Position	Identified	Position	Identified	Position
Main	×	36	×	45	✓	2
MazeCell	×	43	×	41	✓	7
MazeModel	✓	5	×	32	✓	5.5
PrimaryFrame	×	34	×	28.5	✓	8
MazeView	✓	1	×	36	✓	4
CellSizeModel	×	48.5	×	50.5	×	27.5
RobotBase	×	48.5	×	56.5	✓	3
LeftWallFollower	✓	15	×	36	×	49.5
RightWallFollower	✓	17	×	36	×	49.5
Tremaux	✓	11	×	28.5	×	36
Floodfill	✓	2	✓	20.5	×	36
BoxTemplate	×	39	✓	6.5	×	36
StraightTemplate	×	39.5	✓	6.5	×	36
CornerTemplate	×	39.5	✓	6.5	×	36
CrossTemplate	×	39.5	✓	6.5	×	36
ZigZagTemplate	×	39.5	✓	6.5	×	36
EditableMazeView	✓	7	✓	10	✓	16.5
MazeTemplate	✓	21	×	40	✓	13.5
Direction	×	48.5	×	54	×	49.5
RobotController	✓	3	✓	22	✓	16.5
RobotModelMaster	✓	8	×	46.5	✓	11
RobotModel	✓	9	×	42.5	✓	11
RobotStep	×	48.5	×	58.5	×	63.5
TemplatePeg	×	48.5	×	53	✓	5.5
PegLocation	N/A	-	×	68	×	68
TemplateWall	N/A	-	×	56.5	✓	9
Corner	N/A	-	×	68	×	68
Recall (%)	45.83%		29.63%		48.15%	
Ranking score	27.19		35.63		27.41	
**Key Classes**	**MinClass**		**PageRank**		**ICOOK**	
	**Identified**	**Position**	**Identified**	**Position**	**Identified**	**Position**
Main	✓	10	✓	5	✓	16
MazeCell	✓	1	✓	20	✓	1
MazeModel	✓	6	✓	21	✓	3
PrimaryFrame	✓	12	✓	1	✓	17
MazeView	✓	8	✓	15	✓	2
CellSizeModel	✓	2	×	47	✓	8
RobotBase	✓	7	✓	7	✓	6
LeftWallFollower	×	25.5	×	60.5	×	54
RightWallFollower	×	25.5	×	60.5	×	52
Tremaux	×	25.5	×	34	✓	23
Floodfill	×	25.5	×	56	✓	5
BoxTemplate	×	57.5	×	38.5	×	32
StraightTemplate	×	57.5	×	38.5	×	38
CornerTemplate	×	57.5	×	38.5	×	37
CrossTemplate	×	57.5	×	38.5	×	34
ZigZagTemplate	×	57.5	×	38.5	×	35.5
EditableMazeView	×	33	×	27	✓	9
MazeTemplate	✓	14	✓	23	✓	18
Direction	✓	19	×	58	×	49
RobotController	✓	20	×	26	✓	11
RobotModelMaster	✓	9	✓	18	✓	10
RobotModel	✓	5	✓	14	✓	7
RobotStep	×	63	×	64	×	65.5
TemplatePeg	✓	3	✓	19	✓	21
PegLocation	×	67	×	68	×	68
TemplateWall	✓	4	✓	24	×	28
Corner	×	67	×	68	×	68
Recall (%)	**51.85%**		40.74%		**55.56%**	
Ranking score	27.39		34.39		**26.59**	
**Key Classes**	**h-Index**		**Coreness**			
	**Identified**	**Position**	**Identified**	**Position**		
Main	✓	6.5	✓	6.5		
MazeCell	✓	2.5	✓	18.5		
MazeModel	✓	2.5	✓	6.5		
PrimaryFrame	✓	16	✓	18.5		
MazeView	✓	6.5	✓	6.5		
CellSizeModel	✓	16	✓	18.5		
RobotBase	✓	16	✓	18.5		
LeftWallFollower	×	53	×	54		
RightWallFollower	×	53	×	54		
Tremaux	×	45	×	39.5		
Floodfill	×	38.5	×	39.5		
BoxTemplate	×	29	×	30.5		
StraightTemplate	×	29	×	30.5		
CornerTemplate	×	29	×	30.5		
CrossTemplate	×	29	×	30.5		
ZigZagTemplate	×	29	×	30.5		
EditableMazeView	✓	16	✓	6.5		
MazeTemplate	✓	6.5	✓	6.5		
Direction	×	45	×	46		
RobotController	✓	6.5	✓	6.5		
RobotModelMaster	✓	16	✓	18.5		
RobotModel	×	29	×	30.5		
RobotStep	×	62	×	63		
TemplatePeg	✓	16	✓	6.5		
PegLocation	×	68	×	68		
TemplateWall	×	29	✓	18.5		
Corner	×	68	×	68		
Recall (%)	44.44%		48.15%			
Ranking score	28.24		38.57			

The bold denotes the best performance value for the Recall and Ranking Score of Maze.

## Data Availability

Not applicable.
